# Thrombocytopenia and insufficient thrombopoietin production in human small-for-gestational-age infants

**DOI:** 10.1038/s41390-022-02107-7

**Published:** 2022-05-14

**Authors:** Satoru Takeshita, Hiroki Kakita, Shimpei Asai, Takafumi Asai, Mari Mori, Hiroko Ueda, Hiromasa Aoki, Mineyoshi Aoyama, Yasumasa Yamada

**Affiliations:** 1 Department of Perinatal and Neonatal Medicine, Aichi Medical University, 1-1 Yazakokarimata, Nagakute, Aichi 480-1195 Japan; 2 Department of Pathobiology, Nagoya City University Graduate School of Pharmaceutical Sciences, 3-1 Tanabedori, Mizuho-ku, Nagoya, Aichi 467-8603 Japan

## Abstract

**Background:**

Small-for-gestational-age (SGA) infants are at increased risk for transient thrombocytopenia. The aim of this study was to determine whether thrombocytopenia in human SGA infants is due to insufficient thrombopoietin (TPO) production.

**Methods:**

A prospective study of 202 infants with gestational age less than 37 weeks was conducted; 30 of them were SGA infants, and 172 were non-SGA infants. Thrombocytopenia was seen in 17 of 30 SGA infants and 40 of 172 non-SGA infants.

**Results:**

Platelet counts were significantly lower in the SGA group than in the non-SGA group at the time of the lowest platelet count within 72 h of birth. The platelet count and immature platelet fraction (IPF) were negatively correlated in non-SGA infants, but not in SGA infants. In addition, the platelet count and TPO were negatively correlated in non-SGA infants. IPF and TPO were significantly lower in SGA than in non-SGA infants with thrombocytopenia.

**Conclusion:**

IPF increased with thrombocytopenia to promote platelet production in non-SGA infants due to increasing TPO, but not in SGA infants. This study found an association between insufficient TPO production and thrombocytopenia in SGA infants. In addition, this study is important for understanding the etiology of thrombocytopenia in SGA infants.

**Impact:**

- The immature platelet fraction was low, and serum thrombopoietin was not increased in small-for-gestational-age (SGA) infants with thrombocytopenia.
- Thrombocytopenia in SGA infants is due to insufficient thrombopoietin production.
- This study is important for understanding the etiology of thrombocytopenia in SGA infants.

## Introduction

Intrauterine growth restriction (IUGR) occurs in approximately 15% of births worldwide.^1,2^ IUGR is characterized by a restrictive environment that prevents the fetus from meeting its genetic potential for growth, and it occurs often in infants who are small for gestational age (SGA).^2^ SGA is defined as a birth weight of less than the 10th percentile for gestational age.^3^ SGA infants are at increased risk of transient thrombocytopenia.^3,4^ Some reports showed that 31–53% of SGA infants developed thrombocytopenia, generally defined as a platelet count less than 150 × 10^3^/µL, within the first week after birth.^3,4^ Christensen et al. reported that, in SGA infants with thrombocytopenia, the lowest platelet counts were typically on day 4, with a mean nadir of 93 × 10^3^/µL, and that the platelet count increased to ≥150 × 10^3^/µL by day 14 in half of infants.^3^

The cause of thrombocytopenia in SGA infants has been postulated to be a decrease in platelet production.^5–8^ Sola et al. reported that thrombocytopenic SGA infants had low TPO concentrations and decreased marrow megakaryocytes.^6^ Murray et al. studied circulating burst-forming unit-megakaryocytes/colony-forming unit-megakaryocytes, total cultured megakaryocyte precursors, and mature megakaryocytes in most of the preterm infants with thrombocytopenia who were growth restricted.^7^ They suggested that the abnormal hematological characteristics of newborns with intrauterine growth retardation are a consequence of dysregulation of fetal hemopoiesis occurring proximal to committed megakaryocyte and neutrophil progenitors, most likely at the level of the primitive multipotent hemopoietic stem cell.^7^ Watts et al. reported that platelet counts and megakaryocyte numbers were significantly lower in premature infants than in controls on day 1, and TPO levels at the platelet nadir were significantly lower in neonates than in children.^8^ They suggested that preterm infants have an impaired TPO response to thrombocytopenia. We have previously reported animal studies that showed that chronic hypoxia in utero causes immaturity of liver function and a decrease in TPO expression in the liver, which in turn suppresses platelet production.^9^ However, the etiology of thrombocytopenia in human SGA infants remains unclear. The present study attempted to demonstrate that thrombocytopenia in human SGA infants is due to insufficient TPO production.

## Methods

**Fig. 1:**
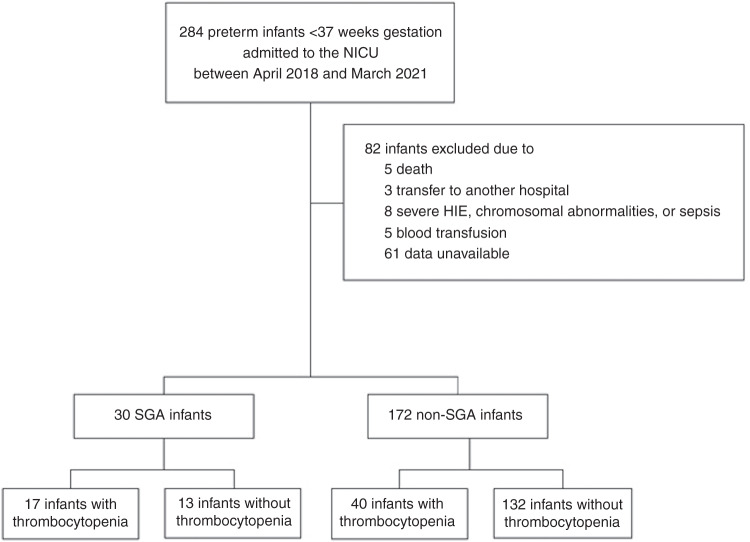
Study profile. SGA small-for-gestational-age.

A prospective study of infants admitted to the Aichi Medical University Hospital neonatal intensive care unit was performed. Clinical data from all infants with gestational age less than 37 weeks, born between April 2018 and March 2021, were gathered. Infants with chromosomal abnormalities, neonatal death, hypoxic-ischemic encephalopathy, congenital malformation syndrome, received blood transfusion, maternal-fetal transfusion syndrome, sepsis, suspected fetal infection, or no available data were excluded. SGA infants were defined as those whose weight at birth was less than the 10th percentile, and non-SGA infants were defined as those whose weight at birth was greater than the 10th percentile. Thrombocytopenia was defined as a platelet count less than 150 × 10^3^/µL. A total of 202 infants were enrolled during the study period (Fig. 1); 30 were SGA infants and 172 were non-SGA infants (Fig. 1), and 17 of 30 SGA infants and 40 of 172 non-SGA infants showed thrombocytopenia (Fig. 1). This study was approved by the ethics committee of Aichi Medical University Hospital. Written, informed consent was obtained from a parent.

Laboratory data, including the white blood cell (WBC) count, hemoglobin (Hb), platelet count, and IPF at the time of the lowest platelet count within 72 h after birth, were collected. The platelet counts at 7 and 14 days of age were also collected. Serum TPO was measured using ELISA (Human Thrombopoietin ELISA Kit, R&D Systems Inc., Minneapolis, MN) in infants with thrombocytopenia at the time of the lowest platelet count within 72 h after birth.

All statistical analyses were performed using EZR (Saitama Medical Center, Jichi Medical University, Saitama, Japan), a graphical user interface for R software (The R Foundation for Statistical Computing, Vienna, Austria). For parametric variables, Student’s *t*-test was used, whereas Fisher’s exact test was used for categorical variables. Coefficients of correlation were tested using Pearson’s two-tailed test. Data are reported as means (interquartile range). Statistical significance was set at *p* < 0.05.

## Results

Factors contributing to SGA were hypertensive disorder of pregnancy (HDP) (11/30), umbilical cord factors including limbic attachment (9/30), twin or triplet gestation (7/30), and unknown (3/30). Table 1 shows the infants’ characteristics and a comparison between the SGA and non-SGA infants at birth. There were no significant differences in gestational age, head circumference, the ratios of males and cesarean sections, Apgar score, cord blood pH, HCO_3_-, and base excess between SGA and non-SGA infants. Bodyweight and length were significantly lower in SGA infants than in non-SGA infants, although the head circumferences were not different and greater than the 10th percentile in all SGA infants. In both infant groups, there were no serious complications with thrombocytopenia, such as cerebral and pulmonary hemorrhages.

**Table 1:** Characteristics of all infants.

	Total (*n* = 202)		SGA (*n* = 30)		Non-SGA (*n* = 172)		*p* value
Gestational age (weeks)^a^	32.4	(31.0–35.0)	32.6	(31.2–35.0)	32.4	(30.6–35.0)	ns
Bodyweight (g)^a^	1582 –0.52 SD	(1299–2009) (–1.63 to 0.30)	1389 –2.10 SD	(1132–1511) (–2.51 to 1.41)	1828 0.12 SD	(1426–2194) (–0.47 to 0.74)	<0.05
Length (cm)^a^	38.5 –0.73 SD	(36.0–41.3) (–1.49 to 0.26)	37.5 –1.84 SD	(35.4–39.0) (–2.26 to –1.24)	40.5 0.24 SD	(37.9–42.5) (–0.54 to 0.65)	<0.05
Head circumference (cm)^a^	28.5 –0.57 SD	(26.5–30.0) (–0.83 to 0.36)	28.5 –0.77 SD	(27.0–29.5) (–1.04 to –0.57)	29.4 0.41 SD	(26.6–30.5) (–0.48 to 0.80)	ns
Male^b^	106 (52.4%)		13 (43.3%)		93 (54.1%)		ns
Cesarean section^b^	161 (79.7%)		26 (86.7%)		135 (78.5%)		ns
Apgar score (1‘)^a^	7	(5–8)	7	(5–9)	7	(5–8)	ns
Apgar score (5’)^a^	8	(7–10)	9	(7–10)	8	(7–9)	ns
pH^a^	7.324	(7.285–7.360)	7.316	(7.268–7.343)	7.341	(7.307–7.366)	ns
HCO_3_- (mmol/L)^a^	22.8	(21.0–25.1)	22.4	(21.3–24.3)	23.1	(20.8–25.2)	ns
BE (mmol/L)^a^	–2.9	(–5.0 to –1.1)	–3.6	(–5.4 to –2.2)	–2.2	(–4.7 to –0.7)	ns

*ns* not significant.

^a^Values are shown as median (interquartile range).

^b^Values are shown as numbers (%).

**Fig. 2:**
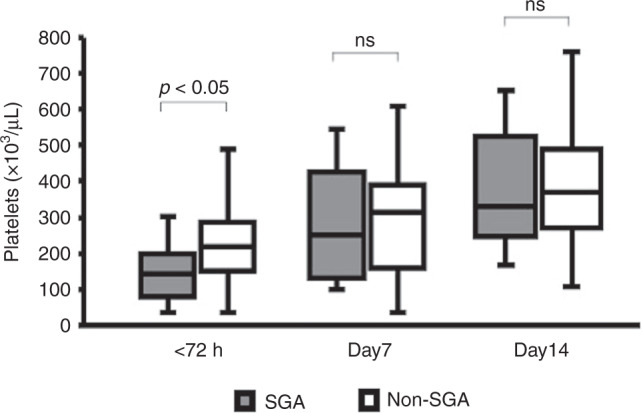
Time course of platelet counts at the time of the lowest platelet count within 72 h after birth in all infants. The figure shows box plots with whiskers. The horizontal line indicates the median. The box indicates the interquartile range. The whiskers indicate the full range. SGA small-for-gestational-age, ns not significant.

**Fig. 3:**
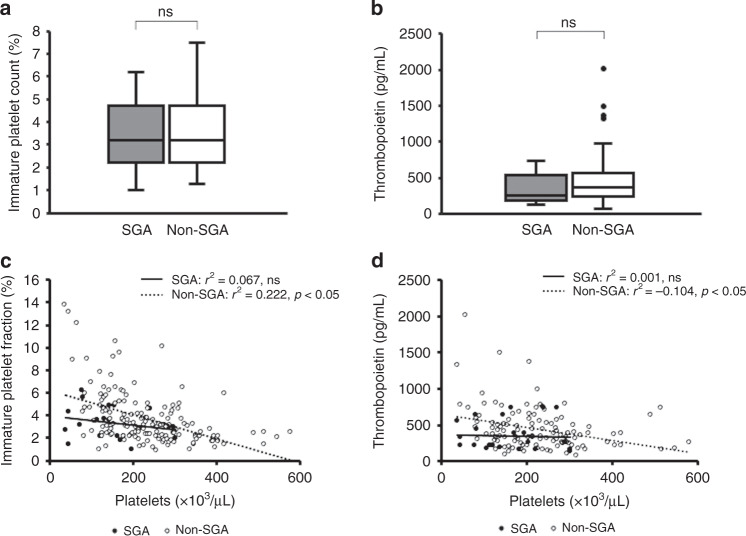
Immature platelet fraction and thrombopoietin at the time of the lowest platelet count within 72 h after birth in all infants and the coefficients of correlation with the platelet count. The figure shows box plots with whiskers. The horizontal line indicates the median. The box indicates the interquartile range. The whiskers indicate the full range. SGA small-for-gestational-age, ns not significant. **a** Immature platelet fraction at the time of the lowest platelet count within 72 h after birth. **b** Thrombopoietin at the time of the lowest platelet count within 72 h after birth. **c** Coefficients of correlation between the platelet count and immature platelet fraction. **d** Coefficients of correlation between the platelet count and thrombopoietin.

Table 2 shows the WBC count, Hb, and platelet count values at the time of the lowest platelet count within 72 h after birth in each group. There was no significant difference in the WBC count at the time of the lowest platelet count within 72 h after birth between the two groups (SGA: 10,100 (7600–13,100)/µL, non-SGA: 10,200 (7100–11,200)/µL, ns). Hb was significantly higher in SGA infants than in non-SGA infants (SGA: 19.9 (17.7–21.0)/g/dL, non-SGA: 16.5 (13.9–18.3) g/dL, *p* < 0.05). The platelet count was significantly lower in SGA infants than in non-SGA infants at the time of the lowest platelet count within 72 h after birth (SGA: 150 (92–216) ×10^3^/µL, non-SGA: 233 (181–294) ×10^3^/µL, *p* < 0.05), but not after 7 days of age (Table 2 and Fig. 2). There were no significant differences in IPF and TPO at the time of the lowest platelet count within 72 h after birth (IPF, SGA 3.2 (2.2–4.6)%, non-SGA 3.2 (2.5–4.7)%, ns; TPO, SGA 330 (167–690), non-SGA 470 (216–712) pg/mL, ns) (Fig. 3a, b). However, the platelet count and IPF were negatively correlated in non-SGA infants (*r*^2^ = 0.222, *p* < 0.05), but not in SGA infants (*r*^2^ = 0.067, ns) (Fig. 3c). In addition, the platelet count and TPO were also negatively correlated in non-SGA infants (*r*^2^ = 0.104, *p* < 0.05), but not in SGA infants (*r*^2^ = 0.001, ns) (Fig. 3d). IPF and TPO have been reported to be useful markers for identifying the cause of thrombocytopenia.^10^

**Table 2:** WBC, Hb, and platelet count at the time of the lowest platelet count within 72 h after birth in all infants.

	Total (*n* = 202)		SGA (*n* = 30)		Non-SGA (*n* = 172)		*p* value
WBC (/µL)	10,100	(7450–12,500)	10,200	(7100–11,200)	10,100	(7600–13,100)	ns
Hb (g/dL)	17.9	(15.5–19.5)	19.9	(17.7–21.0)	16.5	(13.9–18.3)	<0.05
Platelet (×10^3^/µL)	208	(135–281)	150	(92–216)	233	(181–294)	<0.05

Values are shown as median (interquartile range).

*ns* not significant.

Table 3 shows the infants’ characteristics and a comparison between the SGA and non-SGA infants with thrombocytopenia. The etiology of thrombocytopenia in non-SGA infants was unknown, except in three infants with maternal idiopathic thrombocytopenia. There were also no significant differences, except in bodyweight and length, between SGA and non-SGA infants.

**Table 3:** Characteristics of infants with thrombocytopenia.

	Total (*n* = 57)		SGA (*n* = 17)		Non-SGA (*n* = 40)		*p* value
Gestational age (weeks)^a^	32.0	(29.4–33.7)	32.4	(31.1–33.7)	30.0	(28.5–35.4)	ns
Bodyweight (g)^a^	1429 –1.64 SD	(1260–1740) (–2.28 to –0.32)	1384 –2.24 SD	(1176–1440) (–2.28 to –0.67)	1723 –1.08 SD	(1391–1898) (–2.15 to –0.15)	<0.05
Length (cm)^a^	37.5 –1.44 SD	(36.0–38.8) (–2.20 to –0.73)	37.0 –2.20 SD	(36.0–37.5) (–2.29 to –1.93)	38.5 –0.73 SD	(36.5–40.1) (–1.35 to 0.50)	<0.05
Head circumference (cm)^a^	27.8 –0.72 SD	(26.0–29.5) (–0.91 to –0.08)	27.8 –0.91 SD	(26.0–29.1) (–1.24 to –0.85)	28.1 0.07 SD	(26.0–30.6) (–0.15 to 0.33)	ns
Male^b^	23 (40.4%)		8 (47.1%)		17 (42.5%)		ns
Cesarean section^b^	29 (50.9%)		9 (52.9%)		20 (50.0%)		ns
Apgar score (1‘)^a^	7	(4–8)	7	(4–7.5)	7	(4.75–9)	ns
Apgar score (5’)^a^	8	(7–10)	9	(7–10)	7.5	(6.5–8.5)	ns
pH^a^	7.333	(7.247–7.384)	7.319	(7.182–7.364)	7.363	(7.278–7.395)	ns
HCO_3_^-^ (mmol/L)^a^	23.4	(21.5–25.4)	21.8	(18.6–24.8)	24.5	(22.4–28.2)	ns
BE (mmol/L)^a^	–2.8	(–6.7 to –1.1)	–4.5	(–5.1 to –0.9)	–1.8	(–2.7 to 0.80)	ns

*ns* not significant.

^a^Values are shown as median (interquartile range).

^b^Values are shown as numbers (%).

**Fig. 4:**
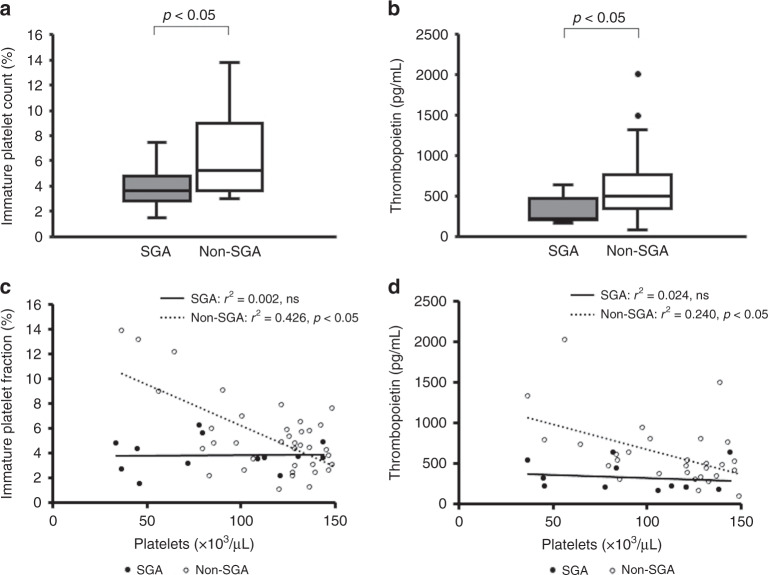
Immature platelet fraction and thrombopoietin at the time of the lowest platelet count within 72 h after birth in infants with thrombocytopenia and the coefficients of correlation with the platelet count. The figure shows box plots with whiskers. The horizontal line indicates the median. The box indicates the interquartile range. The whiskers indicate the full range. SGA small-for-gestational-age, ns not significant. **a** Immature platelet fraction at the time of the lowest platelet count within 72 h after birth. **b** Thrombopoietin at the time of the lowest platelet count within 72 h after birth. **c** Coefficients of correlation between the platelet count and the immature platelet fraction. **d** Coefficients of correlation between the platelet count and thrombopoietin.

Table 4 shows the WBC count, Hb, and platelet count at the time of the lowest platelet count within 72 h after birth in the two groups with thrombocytopenia. There was no significant difference in the WBC count at the time of the lowest platelet count within 72 h after birth between the two groups with thrombocytopenia (SGA: 9600 (6500–11,400)/µL, non-SGA: 11,200 (7700–16,000)/µL, ns) (Table 4). Hb was significantly higher in SGA infants than in non-SGA infants with thrombocytopenia (SGA: 19.3 (16.7–20.5)/g/dL, non-SGA: 16.7 (13.9–20.8) g/dL: *p* < 0.05). The platelet count was significantly lower in SGA infants than in non-SGA infants (SGA: 81 (51–119) ×10^3^/µL, non-SGA: 127 (100–137) ×10^3^/µL, *p* < 0.05) (Table 4). IPF was significantly lower in SGA infants than in non-SGA infants with thrombocytopenia (SGA: 3.7 (3.0–4.8)%, non-SGA: 5.3 (3.7–8.9)%, *p* < 0.05) (Fig. 4a). Similar to IPF, TPO was significantly lower in SGA infants than in non-SGA infants with thrombocytopenia (SGA: 219 (180–322) pg/ml, non-SGA: 554 (328–897) pg/mL, *p* < 0.05) (Fig. 4b). In addition, the correlations of the platelet count with IPF and TPO were investigated in the two groups with thrombocytopenia. There were negative correlations in non-SGA infants with thrombocytopenia, but not in SGA infants with thrombocytopenia, between the platelet count and IPF (non-SGA: *r*^2^ = 0.426, *p* < 0.05, SGA: *r*^2^ = 0.002, ns), and between the platelet count and TPO (non-SGA: *r*^2^ = 0.240, *p* < 0.05, SGA: *r*^2^ = 0.024, ns) (Fig. 4c, d). Together, these results showed that IPF increased with thrombocytopenia to promote platelet production in non-SGA infants due to increasing TPO, but not in SGA infants.

**Table 4:** WBC, Hb, and platelet count at the time of the lowest platelet count within 72 h after birth in infants with thrombocytopenia.

	Total (*n* = 57)		SGA (*n* = 17)		Non-SGA (*n* = 40)		*p* value
WBC (/µL)	10,500	(6600–13,200)	9600	(6500–1400)	11,200	(7700–16,000)	ns
Hb (g/dL)	17.7	(15.0–20.8)	19.3	(16.7–20.5)	16.7	(13.9–20.8)	<0.05
Platelet (×10^3^/µL)	121	(83–133)	81	(51–119)	127	(100–137)	<0.05

Values are shown as median (interquartile range).

*ns* not significant.

## Discussion

The present study demonstrated that preterm SGA infants had significantly lower platelet counts, and that the platelet count and IPF were negatively correlated in non-SGA infants, but not in SGA infants. In addition, the platelet count and TPO were negatively correlated in non-SGA infants, but not in SGA infants. It was also demonstrated that SGA infants with thrombocytopenia had lower IPF and serum TPO than non-SGA infants. These results suggest that thrombocytopenia in SGA infants is due to insufficient TPO production. These findings are important for understanding the etiology of thrombocytopenia in SGA infants.

SGA is caused by maternal factors such as HDP, malnutrition, psychosocial stress, and smoking, as well as neonatal factors such as multiple births and congenital diseases.^11–14^ The risk of developing HDP increases with maternal age and thinness in Japan.^15^ In this study, the head circumferences were greater than the 10th percentile in all SGA infants. These results demonstrate that all SGA infants showed asymmetrical growth due to maternal/placental factors.^9,12^ These SGA infants are at increased risk for complications, such as prematurity, asphyxia, hypothermia, hypoglycemia, hypocalcemia, polycythemia, and thrombocytopenia.^1,2^ Chronic intrauterine hypoxia caused by maternal factors for SGA infants leads to high fetal erythropoietin and polycythemia.^1,2^ In the present study, similar to the previous studies, Hb was significantly higher in SGA infants than in non-SGA infants.^3,4^ The etiology of thrombocytopenia in SGA infants remains unknown. Thus, it is crucial to clarify the etiology and management of thrombocytopenia in SGA infants.

TPO is a major physiological regulator protein that promotes the differentiation and proliferation of megakaryocytes to platelets.^16^ TPO is produced constantly in the liver and binds to the TPO receptor on the surface of megakaryocytic cells, promoting production of platelets via signaling pathways such as JAK-STAT and RAS-MAPK.^17,18^ The main causes of thrombocytopenia are increasing destruction/consumption of circulating platelets and decreased platelet production in the bone marrow.^10^ The cause of thrombocytopenia in SGA infants has been postulated to be a decrease in platelet production.^5–9,19^ However, there are few studies on infants, and the etiology of thrombocytopenia in human SGA infants remains unclear. Wasiuk et al. evaluated thrombopoiesis in SGA infants and postulated that intrauterine hypoxia is responsible for the increase of erythropoietin and impairment of thrombopoiesis in SGA infants.^5^ However, they did not compare TPO levels between SGA and non-SGA infants. Amariyo et al. reported that TPO and inflammatory cytokine levels in cord blood samples from SGA infants were significantly higher than in appropriate-for-gestational-age infants.^20^ They suggested that this increase was caused by a state of inflammation in the IUGR fetus.^20^ In the present study, the timing of blood sampling was later than in their study, and this discrepancy between study findings might be due to this difference in timing.

We previously demonstrated that a decrease in TPO production due to hepatic dysmaturation resulted in thrombocytopenia in SGA model rats.^9^ In the present study, SGA infants with thrombocytopenia had lower IPF and serum TPO levels than non-SGA infants with thrombocytopenia. These results suggest that IPF and TPO levels do not increase in response to thrombocytopenia in human SGA infants, similar to SGA model rats. This is the first report to show human infant data in agreement with those of our animal experiments.

The brain is symmetrically smaller in SGA infants of fetal origin, whereas the brain is protected by the brain-sparing effect in SGA infants of non-fetal origin.^21,22^ In the present study, the length and weight of SGA infants were lower than those of non-SGA infants, but the head circumference was not significantly different from that of non-SGA infants. These results suggest that other organs including the liver, rather than the brain, are dysmature in SGA infants. The decrease in TPO production may also be reflected by this liver dysmaturity, because TPO is mainly produced in the liver, which is susceptible to hypoxia.

There are some limitations to this study. It was a prospective study in a single hospital with a limited number of patients. Biases in patients’ background characteristics and treatment strategies may also have been present. In the present study, most SGA infants were born at a gestational age of more than 30 weeks. The lack of serious complications with thrombocytopenia might have been due to them being fairly mature infants. In addition, whether early administration of a TPO receptor agonist was effective for thrombocytopenia and improved the prognosis in SGA infants was not investigated.

In conclusion, thrombocytopenia in SGA infants could be due to insufficient platelet production caused by a decrease in TPO levels. These results are consistent with previous studies^5–8^ and are important for understanding the etiology of thrombocytopenia in SGA infants.

## Data Availability

The datasets generated during and/or analyzed during the current study are available from the corresponding author on reasonable request.

## References

[1] Kramer MS (2003). The epidemiology of adverse pregnancy outcome: an overview. J. Nutr..

[2] Sankaran S, Kyle PM (2009). Aetiology and pathogenesis of IUGR. Best. Pract. Res. Clin. Obstet. Gynaecol..

[3] Christensen RD (2015). Thrombocytopenia in small for gestational age infants. Pediatr.

[4] Fustolo-Gunnink SF (2016). Early-onset thrombocytopenia in small-for-gestational-age neonates: a retrospective cohort study. Plos One.

[5] Wasiluk A (2009). Thrombopoiesis in small for gestational age newborns. Platelets.

[6] Sola MC, Calhoun DA, Hutson AD, Christensen RD (1999). Plasma thrombopoietin concentrations in thrombocytopenic and non‐thrombocytopenic patients in a neonatal intensive care unit. Br. J. Haematol..

[7] Murray NA, Roberts IA (1996). Circulating megakaryocytes and their progenitors in early thrombocytopenia in preterm neonates. Pediatr. Res..

[8] Watts TL, Murray NA, Roberts IA (1999). Thrombopoietin has a primary role in the regulation of platelet production in preterm babies. Pediatr. Res..

[9] Takeshita S (2021). Insufficient thrombopoietin due to hepatic dysmature results in thrombocytopenia in small-for-gestational-age rats. Br. J. Haematol..

[10] Jeon K (2020). Immature platelet fraction: a useful marker for identifying the cause of thrombocytopenia and predicting platelet recovery. Medicine.

[11] Cheng J, Li J, Tang X (2020). Analysis of perinatal risk factors for small-for-gestational-age and appropriate-for-gestational-age late-term infants. Exp. Ther. Med..

[12] Berger H (2020). Impact of diabetes, obesity and hypertension on preterm birth: population-based study. PloS One.

[13] Hobel C, Culhane J (2003). Role of psychosocial and nutritional stress on poor pregnancy outcome. J. Nutr..

[14] Vrijkotte TG, van der Wal MF, van Eijsden M, Bonsel GJ (2009). First-trimester working conditions and birthweight: a prospective cohort study. Am. J. Public Health.

[15] Takemoto Y, Ota E, Yoneoka D, Mori R, Takeda S (2016). Japanese secular trends in birthweight and the prevalence of low birthweight infants during the last three decades: a population-based study. Sci. Rep..

[16] Kato T (1998). Native thrombopoietin: structure and function. Stem Cells.

[17] Royer Y, Staerk J, Costuleanu M, Courtoy PJ, Constantinescu N (2005). Janus kinases affect thrombopoietin receptor cell surface localization and stability. J. Biol. Chemi.

[18] Kuter DJ (2013). The biology of thrombopoietin and thrombopoietin receptor agonists. Int. J. Hematol..

[19] Cremer M, Weimann A, Hammer H, Bührer C, Dame C (2010). Immature platelet values indicate impaired megakaryopoietic activity in neonatal early-onset thrombocytopenia. Thromb. Haemost..

[20] Amarilyo G (2011). Increased cord serum inflammatory markers in small-for gestational-age neonates. J. Perinatol..

[21] Roza SJ (2008). What is spared by fetal brain-sparing? Fetal circulatory redistribution and behavioral problems in the general population. Am. J. Epidemiol..

[22] Simanaviciute D, Gudmundsson S (2006). Fetal middle cerebral to uterine artery pulsatility index ratios in normal and pre-eclamptic pregnancies. Ultrasound Obstet. Gynecol..

